# Unraveling the microRNAs Involved in Fasciolosis: Master Regulators of the Host–Parasite Crosstalk

**DOI:** 10.3390/ijms26010204

**Published:** 2024-12-29

**Authors:** Diana María Barrero-Torres, Guillem Herrera-Torres, José Pérez, Álvaro Martínez-Moreno, Francisco Javier Martínez-Moreno, Luis Miguel Flores-Velázquez, Leandro Buffoni, Pablo José Rufino-Moya, María Teresa Ruiz-Campillo, Verónica Molina-Hernández

**Affiliations:** 1 Departamento de Anatomía y Anatomía Patológica Comparadas y Toxicología, UIC Zoonosis y Enfermedades Emergentes (ENZOEM), Universidad de Córdoba, Campus de Rabanales, Edificio Sanidad Animal, 14071 Córdoba, Spain; v82bbtod@uco.es (D.M.B.-T.); v82hetog@uco.es (G.H.-T.); an1pearj@uco.es (J.P.); luis.floresv@uss.cl (L.M.F.-V.); 2 Departamento de Sanidad Animal (Área de Parasitología), UIC Zoonosis y Enfermedades Emergentes (ENZOEM), Universidad de Córdoba, Campus de Rabanales, Edificio Sanidad Animal, 14071 Córdoba, Spain; amm@uco.es (Á.M.-M.); fjmartinez@uco.es (F.J.M.-M.); h12bupel@uco.es (L.B.); v32rumop@uco.es (P.J.R.-M.); 3 Unidad de Anatomía, Histología y Patología Veterinaria, Escuela de Medicina Veterinaria, Facultad de Ciencias Naturales, Universidad San Sebastián, Campus Puerto Montt, Puerto Montt 5480000, Chile

**Keywords:** microRNAs (miRNAs), miRNome, *Fasciola hepatica*, *Fasciola gigantica*, zoonosis, pathogenesis, non-coding RNAs (ncRNAs), miRBase, immunity

## Abstract

Fasciolosis is a neglected tropical disease caused by helminth parasites of the genus *Fasciola* spp., including *Fasciola hepatica* (*F. hepatica*) and *Fasciola gigantica* (*F. gigantica*), being a major zoonotic problem of human and animal health. Its control with antihelminthics is becoming ineffective due to the increase in parasite resistance. Developing new therapeutic protocols is crucial to a deeper knowledge of the molecular bases in the host–parasite interactions. The high-throughput omics technologies have dramatically provided unprecedented insights into the complexity of the molecular host–parasite crosstalk. MicroRNAs (miRNAs) are key players as critical regulators in numerous biological processes, modifying the gene expression of cells by degradation of messenger RNA (mRNA), regulating transcription and translation functions, protein positioning, cell cycle integrity, differentiation and apoptosis. The large-scale exploration of miRNAs, including the miRNome, has offered great scientific knowledge of steps in fasciolosis, further scrutinizing the pathogenesis, the growth and development of their strains and their interaction with the host for the survival of the different parasite stages. This review compiles the updated knowledge related to miRNAs involved in fasciolosis and the generated miRNome, highlighting the importance of these key molecules in the host–parasite interactions and the pathogenesis of *Fasciola* spp. directing towards the development of new biotherapeutic protocols for the control of fasciolosis.

## 1. Introduction

Fasciolosis is part of the neglected tropical diseases cataloged by the World Health Organization (WHO) [1]. It is caused by parasites of the genus *Fasciola* spp., which includes the species of *Fasciola hepatica* (*F. hepatica*) and *Fasciola gigantica* (*F. gigantica*) and has been described as the most geographically widespread parasitic zoonosis [2,3] with more than 180 million people at risk of infection [4]. Fasciolosis is a major problem in the farming industry as it is responsible for low fertility and a reduction in milk and meat production in sheep, goats and cattle, causing economic losses estimated at USD 3.2 billion per year [5].

The disease control system is based on the rotation of paddocks and the use of antihelminthics such as triclabendazole (TCBZ) [6]. However, the widespread use of TCBZ has led to a reduction in the efficacy due to the development of parasitic resistance [6,7], adopted by an evolutionary mechanism in the new parasitic strains, starting from the populations that survive the treatment, where a small genomic region was inherited with dominant traits of resistance [8,9].

Infections are recognized for their chronicity due to the successful survival of invaders within the mammalian host [10,11]. *F. hepatica* survival is managed by a series of interactions between the immune system and the excretory/secretory products (ESPs) the parasite releases, some of them provided through extracellular vesicles (EVs) [12,13]. These ESPs are involved in the dysregulation of host genes associated with metabolism, immune response and tissue repair/regeneration [14].

The use of omics has improved information and understanding of the entire *F. hepatica* genome [15,16], evidencing the role of non-coding RNAs (ncRNAs) such as micro-RNAs (miRNAs) in modifying the gene expression of cells by degradation of messenger RNA (mRNA) [17,18], regulating transcription and translation functions, protein positioning, cell cycle integrity, differentiation and apoptosis [19,20]. Small RNAs are a type of non-coding RNA (ncRNA) that includes miRNAs, among others. They are associated with Argonaute family proteins (Ago family proteins) and suppress unwanted genetic materials and transcripts (RNA silencing or RNA interference, RNAi) [21,22,23]. RNAi fragments provide post-transcriptional silencing of proteins, resulting in the genetic domain in a cell type directing its phenotype and functionality, being classified as vital in biological development, homeostasis and alteration in a living organism [24,25].

The large-scale exploration of miRNAs, including the miRNome, has offered great evolutionary steps, revealing the participation of these in processes such as apoptosis, differentiation, cell proliferation, DNA repair, lipid metabolism, enzymes and amino acids, among other functions of cells [26]. This innovation has been key in the interaction of pathogens in a mammalian host, as they are involved in the immune response, which helps to understand the pathogenesis of many metabolic alterations and in the search for new biotherapeutics that help in the control and/or eradication of these [25,27,28].

The miRNA annotation system is based on published experimental investigations of different organisms (animals and plants) that identify sequences of stable loops in the genome. With a mapping system, the stable loops that correctly assemble generate a reliable, mature miRNA [29]. These sequences are integrated into open-access databases such as miRBase.org (Release 22.1), miRCarta (Version 1.1; https://mircarta.cs.uni-saarland.de/, accesed on 24 December 2024), miRGeneDB (Version 2.0; https://mirgenedb.org/ accesed on 24 December 2024) and RumimiR (https://rumimir.sigenae.org/ accesed on 24 December 2024) as repositories to facilitate prediction, validation and annotation of identified target miRNAs and generate a landscape for searching for new sequences [30,31,32,33].

Since the first comparative characterization of miRNAs from *F. gigantica* and *F. hepatica* and the discovery of its implication in the modulation of host immune responses, a better understanding of miRNAs and miRNome in fasciolosis has been postulated as crucial considering that are hypothesized as promising “anchors” in the binding of molecular networks, directing towards new biotherapeutic protocols for the control of this disease [34,35,36,37,38]. Thus, this review presents a current version of the miRNAs identified in fasciolosis and the miRNome derived from its analysis, highlighting the importance of these key molecules in the host–parasite interactions and the pathogenesis of *Fasciola* spp. To achieve this, this narrative review was conducted involving a subjective examination and critique of an entire body of literature related to miRNAs from 1993 to 2024. The most relevant research concerning *Fasciola* spp. was selected to create a comprehensive state-of-the-art review, adhering to the FINER (Feasible, Interesting, Novel, Ethical and Relevant) criteria. Thus, this review outlines the current status of miRNAs, including their discovery and historical milestones, biogenesis, the key miRNAs involved in fasciolosis and its pathogenesis, as well as an overview of the miRNome, while providing insights aimed at advancing the field.

## 2. A Look at the Past in microRNAs: Discovery and Historical Landmarks

The dogma of molecular biology recognizes RNA molecules for their ability to synthetize proteins from the genetic information encoded in the DNA. However, the function of RNA molecules is not limited to being a messenger for protein synthesis, and only about 1–2% of the RNA present within a human cell is protein-coding, the remainder being ncRNA [39].

**Figure 1:**
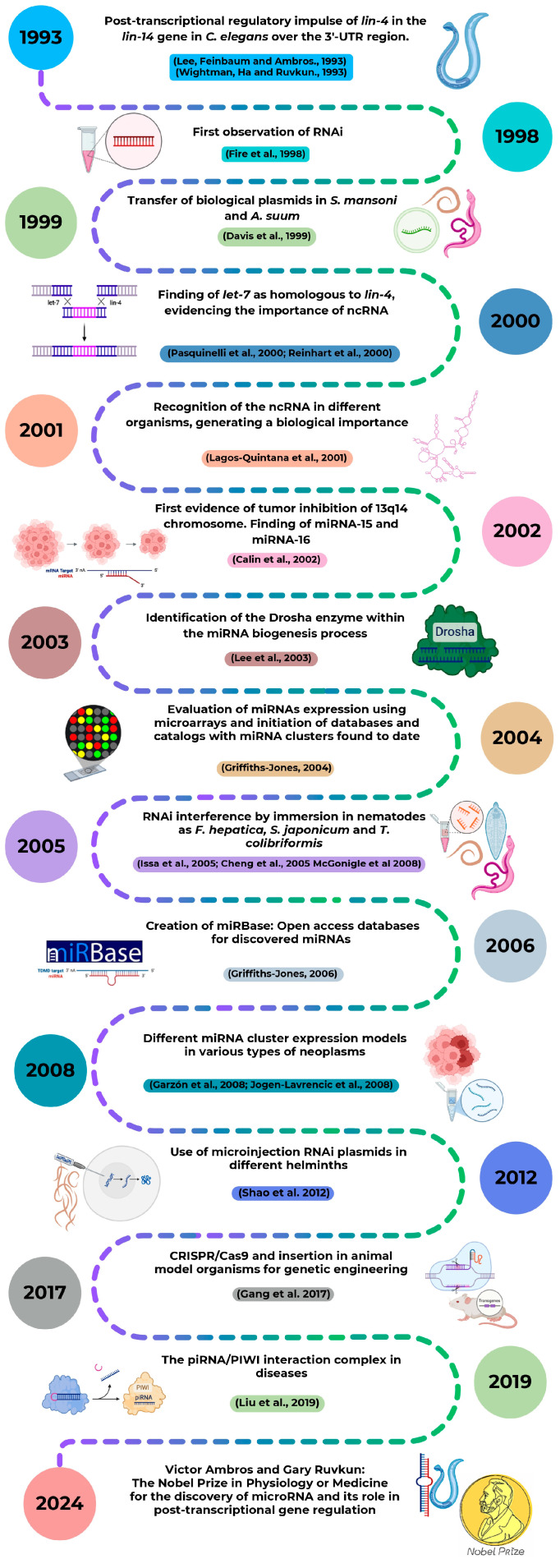
Chronology of microRNA discovery and remarkable historical landmarks [29,41,42,43,44,45,46,47,48,49,50,51,52,53,54,55,56,57,58,59].

The first evidence that not all RNA molecules code for proteins described that the heterochronic *lin-4* gene can interact with the regulatory site of the *lin-14* gene, exerting a negative control leading to suppression of its function [40]. Eventually, the implication of *lin-4* gene in the regulation of nematode larval development in *Caenorhabditis elegans* (*C. elegans*) was demonstrated [41] (Figure 1). Interestingly, *lin-4* does not encode a protein but small transcripts of approximately 22 and 61 nucleotides (nt), which contain sequences complementary to a repeated sequence element in the 3′ untranslated region (3′-UTR) of *lin-14* mRNA, suggesting that *lin-4* regulates *lin-14* translation via an antisense RNA-RNA interaction [41]. In this way, Lee et al. 1993 [41] and Wightman et al. 1993 [42] demonstrated that the gene *lin-4* in *C. elegans* exerts a post-transcriptional regulatory impulse on the gene *lin-14* specifically on the 3′-UTR region that did not code for proteins, being these the first miRNA discoveries that paved the way to a new gene regulation paradigm where short ncRNAs of 22 nt have the ability to repress mRNA expression by specific binding to the 3′-UTR region [41,42].

Since this first discovery, a serial of crucial findings occurred, enriching the knowledge about miRNAs and the role they play as critical regulators in numerous biological processes (Figure 1). Studies related to the effectiveness of double-stranded RNA compared to single strands at producing interference and RNA transfection experiments in helminth parasites to study gene expression were eventually carried out [43,44]. In 2000, the *let-7* RNA gene and its implication in the timing of *C. elegans* development together with *lin-4* was reported, reaffirming the manipulation exerted by ncRNAs in gene expression [45,46]. Particularly, miRNAs’ post-transcriptional regulatory mechanisms, both in invertebrates and vertebrates, seem to play an important role that should be taken into account in future studies [47]. Being up or downregulated, miRNAs were discovered to be implicated in the development of certain disorders, namely B cell chronic lymphocytic leukemia and acute myeloid leukemia [48,49,50]. The identification of RNase III endonuclease enzyme (Dicer) as the enzyme implicated in the cytoplasmic processing of precursor miRNA (pre-miRNAs) into mature miRNAs was previously described, and the discovery of the Drosha Ribonuclease II (DROSHA) enzyme implication in nuclear processing of the primary miRNA (pri-miRNAs) completed the understanding of the miRNAs biogenesis mechanism [51]. The first sequence and annotation data derived from the discovered miRNAs were published in an accessible database [29], and eventually, the miRBase repository was created, aiming to provide integrated interfaces to comprehensive miRNA sequence data, annotation and predicted gene targets [52].

Advanced techniques such as immersion RNAi [53,54,55] and RNAi microinjection [56] were implemented as gene knock-out strategies that could reduce parasitic infection. The development of a method to target gene disruptions and mutant phenotypes was studied in parasites, introducing Clustered Regularly Interspaced Short Palindromic Repeats associated protein 9 (CRISPR-Cas9)-mediated gene disruptions to enable future studies of gene function [57]. This, together with the discovery of ncRNAs that interact with the PIWI-interacting RNA (piRNA/PIWI complex), which are expressed in a tissue-specific manner, thus participating in various diseases, could become potential targets for therapeutic intervention [58].

The invaluable potential of miRNAs was officially recognized when Victor Ambros and Gary Ruvkun were awarded the 2024 Nobel Prize in Physiology or Medicine for the discovery of microRNA and its role in post-transcriptional gene regulation [59].

## 3. Location and Biogenesis of microRNAs

**Figure 2:**
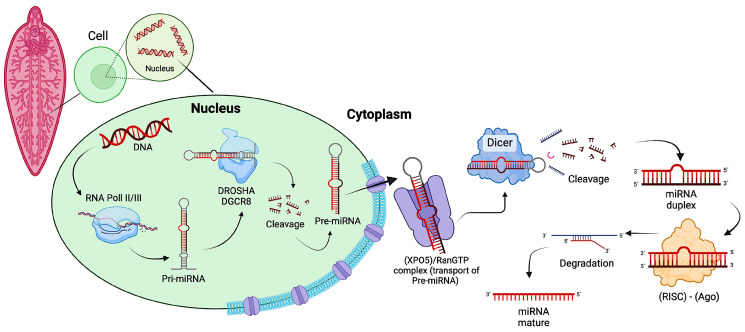
Representation of microRNA (miRNA) biogenesis in *Fasciola hepatica* through the canonical pathway.

The understanding of the location of miRNAs is key to determining how miRNAs are transcriptionally regulated. According to their genomic position, they can be classified into intragenic and intergenic miRNAs. Those located within protein-coding or non-coding genes are called intragenic miRNAs, and genes in which the miRNAs are embedded are called host genes (intergenic) [60,61]. The biogenesis and function of miRNAs are tightly regulated, and their dysregulation is often associated with diseases and disorders [23]. The biogenesis of miRNAs is divided into two important pathways: canonical and non-canonical [62,63]. The canonical is the dominant pathway by which miRNAs are processed (Figure 2). In this, the pri-miRNA is transcribed from its gene and processed into pre-miRNA by the Microprocessor complex, which consists of a DROSHA and an RNA binding protein called DiGeorge syndrome critical region 8 (DGCR8) in vertebrates, which are localized in the nucleus [63,64]. DROSHA is responsible for cleaving the DGCR8-recognized pri-miRNA to the 70 nt pre-miRNA at a sufficient distance from the base of the hairpin, defining one end of the mature miRNA. Once pre-miRNAs are synthesized, an exportin 5 (XPO5)/RanGTP complex transports them to the cytoplasm, and they are processed by the enzyme called Dicer, which further cleaves the pre-miRNA to a miRNA duplex and selects the miRNA with the least stable 50-end for loading into the RNA-induced silencing complex (RISC), whose primary constituent is an Ago protein, which degrades the duplex miRNA to generate a single strand with 3′ and 5′ ends called mature miRNA or miRNA-RISC (miRISC) (Figure 2). The mature miRNA is then capable of targeting mRNA transcripts for repression through a variety of mechanisms [49,65].

In the non-canonical pathway, diverse small RNAs, structurally and functionally like miRNAs, are synthesized [28]. It is classified into three groups: DROSHA/DGCR8-independent, Dicer-independent and terminal uridylyl transferases (TUT) dependent [23,28,66]. miRNAs resulting from the non-canonical biogenesis are low in abundance and poorly conserved, and the vast majority of functional miRNAs follow the canonical pathway for their biogenesis. For this reason, the functional relevance of non-canonical miRNAs should be interpreted with caution [23].

## 4. microRNAs Involved in Fasciolosis and Its Pathogenesis

### 4.1. History of Exploring microRNAs in Fasciola spp.

The first study focused on *F. hepatica* miRNAs and described a very small subset of the actual miRNAs available. The absence of a *F. hepatica* reference genome led Xu et al. 2012 [34] to utilize the genome of *Schistosoma japonicum* and use a small RNA sequencing (RNA-seq) and Basic Local Alignment Search Tool (BLAST) approach, identifying a total of 16 miRNA candidates from *F. hepatica*. Eleven miRNAs were shared by *F. hepatica* and *F. gigantica*, including eight conserved and three novels. The conserved miRNAs all matched perfectly with those from *S. japonicum* in the miRBase database. A total of five *F. hepatica*-specific miRNA sequences were identified, namely fhe-mmu-miR-1957, fhe-miR-novel-01, fhe-miR-novel-08, fhe-miR-novel-07 and fhe-miR-novel-10, representing the first small RNA-seq data for *F. hepatica* adults [34] (Table 1).

Using the pipeline miRCandRef and available genomic sequence data [16], the miRNA complement was increased from 16 to 55 miRNAs (41 conserved and 13 novel), and the production of miRNA-containing EVs by *F. hepatica* adults was confirmed [67]. The metazoan conserved fhe-miR10-P2a (named fhe-miR-125b in miRBase) was the most highly expressed miRNA across all stages and in the EV fraction in the adult stage of *F. hepatica* [38]. In this new 55-miRNAs list, the eight conserved miRNAs identified by Xu et al. 2012 [34] were observed. Nevertheless, the eight novel miRNAs proposed were not found [67], suggesting that they are not genuine miRNAs and thus being removed from the listed miRNAs for *F. hepatica* [37]. In addition, mir-2a-A, bantam, mir-1175, mir-277 and let-7 were found among the 10 most highly expressed molecules in the cellular fraction. On the other hand, mir-125b, mir-87, mir-2a-B, mir-2b-A and mir-1993 were among the 10 most highly expressed in the EV data. Concretely, fhe-miR-87 and fhe-miR-1993 are absent in mammals and fhe-miR-2b-A and fhe-miR-2a-B are orthologs of miR-27, bta-mir-27a, and bta-mir-27b. On the other hand, fhe-miR-125b represented the highest expression in EVs [67]. All the conserved miRNA families previously reported in the adult stage of *F. hepatica* (fhe-miR-125b, fhe-miR-bantam, fhe-let-7c, fhe-miR-277 and fhe-miR-71/miR-2 cluster members) [34] were detected and expressed at very high levels in the newly excysted juveniles (NEJs), being miR125b the most abundantly expressed, followed by let-7 and bantam [68].

With the available full genome of *F. hepatica* [16] and the three small RNA-seq libraries, Fromm et al. 2017 [69] found evidence for the presence of two new miRNA families in *F. hepatica* (mir-210 and mir-750) using improved algorithms. Thus, the miRNA complement of *F. hepatica* consists of 53 members of 32 conserved miRNA families and 5 novel *F. hepatica*-specific miRNA genes. There was evidence that *F. hepatica* miRNA profile varies significantly between adults, juveniles and EVs, and the abundance of the expressed miRNAs also varies through the *F. hepatica* life cycle, indicating stage-specific roles [69]. Concretely, mir-71-P1b is the most expressed miRNA in adult worms, mir-277-p2 is the most expressed in EV data, and mir-10-p2a is the most expressed in juvenile worm-derived samples. The overexpression of Mir-10-p2a, mir-2-p1b, bantam, mir-1175 and mir-190-p2 are the most highly expressed molecules in the cellular fraction, and mir-10-p2b, mir87, mir-2-p2b and mir-2-p4 are among the most highly expressed miRNAs in the EV data [69] (Table 1).

Ovchinnikov et al. 2020 [70] described an increase in the abundance of let-7-P3_5p, mir-10-p2b_5p, mir-83_3p, mir279_3p and mir-1993_3p in EVs relative to the adult worm both in *Schistosoma mansoni (S. mansoni)* and *F. hepatica* and mir-1989_5p was substantially upregulated. In addition, mir-71 and mir-277 families were found to be protostome-specific miRNA, being absent in cows and humans. Moreover, a single nucleotide difference between the mir-277-P2 mature sequence could be interesting to differentiate the two flukes.

Hu et al. 2021 [71] demonstrated that *F. gigantica* and *F. hepatica* showed considerable levels of conservation of miRNAs. A small RNA sequencing of eight life cycle stages of *F. gigantica* was conducted, and 56 miRNAs from 33 conserved families were detected. Out of these, only 10 miRNAs were previously known in *F. gigantica*, being these miRNAs from eight conserved and previously known families (bantam, let-7, two miR-2, miR-10, miR-12, two miR-71, miR-87 and miR124). The top expressed miRNA varied greatly at each stage, evidencing the substantial roles of miRNAs in the parasite’s development. Three of the most highly expressed miRNAs (bantam_3p, miR-71-P1_5p and miR-2-P1a_3p) have also been found in *F. hepatica* belonging to the top expressed miRNAs from NEJs, adults and EVs [69,70] (Table 1).

Fontenla et al. 2022 [38] analyzed the expression of diverse ncRNA in the key stages of invasion and establishment of *F. hepatica* infection in the definitive host. The results indicated that ncRNAs are present in all stages and also in secreted vesicles, miRNAs being the most abundant ncRNA followed by transfer RNA (tRNA)-derived fragments. The comparison with the recent study in *F. gigantica* [71] confirms the presence of 34 conserved families and a growing set of miRNAs described so far only in Fasciolidae in addition to the nine novel miRNAs (fhe-miR-NEW-1 to 9) already described and recompiled recently [37]. The conserved miRNAs most highly expressed across stages and EVs were fhe-miR-125b, fhe-miR-71-P1b and bantam.

A redundant total of 186 mature miRNA sequences was obtained from the manual analysis performed by Herron et al. 2022 [72] of all published *F. hepatica* miRNAs [34,67,68,70]. Naming miRNA sequences was a difficult task as they were found to be inconsistent between individual papers and described with the original miRBase naming system [73]. Small RNA sequencing of multiple *F. hepatica* life stage libraries was obtained, and these datasets were combined and then qualitatively analyzed for miRNAs using miRDeep2, yielding 91 mature miRNA sequences across multiple *F. hepatica* life stages, 29 of which have been previously reported in *F. hepatica* [72] and representing the largest single miRNA dataset reported for *F. hepatica*. Most sequences (61) were newly described miRNAs that lacked matches in miRBase or miRGeneDB searches, providing the first developmental profile of miRNAs in intra-mammalian *F. hepatica* life stages and also providing computational predictions of miRNA—mRNA functional regulatory networks for both cellular and secreted miRNAs, including neuromuscular transcripts, secreted metabolic modulators and nutrient scavengers, secreted proteases and individual components of exosome and glycan biosynthesis with fhe-mir-125a-5p, fhe-mir-1899-5p, fhe-mir-277-3p and fhe-mir-71b-5p were present in all six libraries [72]. 

**Table 1:** The most representative microRNAs (miRNAs) identified in the research on *Fasciola* spp. history.

miRNAs	Target	Reference
fhe-mmu-miR-1957 fhe-miR-novel-01 fhe-miR-novel-08 fhe-miR-novel-07 fhe-miR-novel-10	*Fasciola hepatica*	[34]
mir-2a-A Bantam mir-1175 mir-277 let-7	*F. hepatica*’s cellular fraction	[67]
mir-125b mir-87 mir-2a-B mir-2b-A mir-1993	*F. hepatica*’s extracellular vesicles (EVs)	
miR125b let-7c Bantam miR-277 miR-71/miR-2 (cluster members)	*F. hepatica*’s newly excysted juveniles (NEJs)	[68]
mir-10-p2a mir-2-p1b Bantam mir-1175 mir-190-p2	*F. hepatica*’s cellular fraction	[69]
miR-71-p1b	Overexpression in *F. hepatica*’s adult	
miR-277-p2 EVs	Overexpression in *F. hepatica*’s EVs	
miR-10-p2a NEJs	Overexpression in *F. hepatica*’s NEJs	
mir-10-p2b mir-87 mir-2-p2b mir-2-p4	*F. hepatica*’s EVs	
let-7-P3_5p mir-10-p2b_5p mir-83_3p mir279_3p mir-1993_3p mir-1989_5p (most abundant)	*F. hepatica*’s EVs	[70]
Bantam let-7 miR-2 miR-10 miR-12 miR-71 miR-87 miR124	*Fasciola gigantica*	[71]
fhe-miR-125b fhe-miR-71-P1b Bantam	*F. hepatica*’s EVs	[38]
fhe-mir-125a-5p fhe-mir-1899-5p fhe-mir-277-3p fhe-mir-71b-5p	*F. hepatica*	[72]

### 4.2. Role of microRNAs in Parasite Physiology

The predicted target genes of *F. hepatica* miRNAs are proteins related to reproduction, development processes, response to stimuli, immunomodulation, and locomotion [34]. Most of the miRNAs are highly expressed in metacercariae [72], and the preformation of their vesicles seems to be initiated at that stage [74], where their release in a timely manner may be regulated by fhe-mir-71-P1b, fhe-miR-71-P2, fhe-miR-1-P1, fhe-miR-1-P2, fhe-miR-96, and fhe-miR-7-P1 [38], which are secreted in large amounts by NEJs at different stages of development of *F. hepatica*. This may be related to the induction of stasis in this life stage, where miRNAs could be involved in pausing transcription until a host is encountered. Ricafrente et al. 2022 [18] described that the genome location of most *F. hepatica* miRNAs (70%) was found to be intergenic, and this could be interpreted as a parasite capacity to transcribe their miRNA independently of worm gene expression. This has been recorded as an adaptation in other Platyhelminthes [75,76] to transcribe miRNAs in response to external host signals and regulate host genes without significantly disturbing the parasite’s developmental transcriptome [18].

Herron et al. 2022 [72] were the first to focus on miRNA targets that are demonstrably downregulated during fasciolosis using transcriptomes from sheep/cattle tissues, and 298 miRNA-targeted transcripts were significantly downregulated across fasciolosis transcriptomes from lymph nodes, Peripheral Blood Mononuclear Cells (PBMCs) and liver from sheep and/or cattle. It has been reported that *F. hepatica* cathepsin L3 (FhCL3) expression is switched off as the parasite matures and moves from tissue-feeding to blood-feeding. Interestingly, FhCL3 is the unique target of bantam-3p, and its increase during immature and adult stages may influence the downregulation of FhCL3 expression, suggesting that this protein is specifically required by the NEJs and may play a critical role in the transition from NEJ to the immature worm. Similarly, the SAP30-binding protein regulation is impaired by *F. hepatica* miRNAs in the immature stage and in the adult worms, thus suggesting a critical role in *F. hepatica* egg production in these life stages (Table 2). Another gene highly regulated during the NEJ stage is SACMAD1, suggesting that the miRNA-mediated suppression in NEJs is related to the enhanced proliferation of neoblasts observed during the 24 h after excystment [18].

In NEJs cultured in vivo for 21 days, a downregulation of cathepsin B protease genes together with an upregulation of cathepsin L protease genes have been reported, thus suggesting a switch to produce cathepsin L proteases in *F. hepatica* juveniles in vivo when compared with the NEJs cultured in vitro, which show lower levels of development and are unable to switch cathepsin expression, suggesting that this switch depends on host-specific signals [77]. In addition, the upregulation of fhe-let-7a-5p miRNA and the downregulation of fhe-mir-124-3p in in vivo juveniles is related to cell differentiation, regulation of stem cells, growth induction, and lower levels of neuronal development. Although let-7 and miR-125 have been associated with the same polycistronic transcript and both miRNAs can act together as key regulators of development [78], these miRNAs appear to be organized separately within different scaffolds of the *F. hepatica* genome [18,68].

Overall, in the study by Robb et al. 2022 [77], it is highlighted that, despite the fact that in vitro maintained juveniles are smaller in size and experience a delay in growth and development due to the absence of host signals when compared to the in vivo juveniles, the in vitro maintenance platform covers *F. hepatica* relevant biological processes as >86% of genes are expressed at similar levels in both culture systems (Table 2). In addition, Wnt signaling pathway proteins may have key roles in early-stage developmental processes as an overall downregulation of Wnt-associated mRNAs in in vivo NEJs has been reported [77].

This is how the identification of corresponding gene targets for novel miRNAs discovered across *F. hepatica* three life stages (RNA isolated from NEJs excysted in vitro and cultured for 6 h, 24 h and 7 days) indicates that there is differential expression between the life stages that ensure the biological processes required for each developmental stage and are switched on and off as necessary. Thus, genes associated with the integral component of the membrane appear to be predominant biological processes in the NEJ parasites. As the NEJs develop to immature fluke, overexpression of genes associated with nucleotide synthesis, transcription and translation, energy regulation, cell signaling and proteolysis are found to be overexpressed. Bantam-3p, miR-1b-3p, miR-71a-5p, miR-71c-5p, miR-125b-5p, miR-190a-5p and miR-277-3p were the most abundant miRNAs present in every life stage, changing their relative abundance as the parasite develops, grows and matures [18], as well as miR-126a-3p, miR-150-5p, miR-155-5p, miR-181a-5p and miR-362-3p are activated in an oscillatory manner during parasite proliferation and migration, in conjunction with apoptotic processes in cell differentiation [79].

Taking into account the functionality of miRNAs in Fasciolidae, they have been classified into five expression clusters. Genes targeted to cluster 1 (representing a set of miRNAs highly expressed in metacercariae while lowly expressed in NEJs) are enriched in processes associated with vesicle organization and transport and membrane fusion. Cluster 2 (miRNAs strongly downregulated in metacercariae while upregulated in NEJs and adults) is related to genes implicated in vesicular cargo, maturation and trafficking in the Golgi, IL-12 signaling and response to inorganic substances/temperature stimuli that could be relevant for successful excystment and development. Cluster 3 includes miRNAs highly expressed in adults and is related to cell differentiation and tissue morphogenesis of epithelial cells and cell-to-cell contact-related genes, suggesting its role in the maturation of juvenile forms during the formation of the syncytial tegument. Cluster 4 includes miRNAs highly expressed in NEJs but strongly regulated in adults and functions associated with the production of eggs in the adult stage, like nervous development, transcription regulation, and germinal function. Finally, cluster 5 represents miRNAs strongly downregulated in adults but expressed in metacercariae and NEJs, being associated with vacuolar proton transport ATPases, enzymes that are related to the acidification of vacuoles, a relevant requisite in feeding and interaction with the host [38] (Table 2).

**Table 2:** microRNAs involved in development and biological processes of *Fasciola* spp.

miRNAs	Pathway	Reference
fhe-mir-71-P1b fhe-miR-71-P2 fhe-miR-1-P1 fhe-miR-1-P2 fhe-miR-96 fhe-miR-7-P1	Involved in the timely regulation of the release of *Fasciola hepatica’s* extracellular vesicles (EVs)	[38]
fhe-mir-125a-5p fhe-mir-1899-5p fhe-mir-277-3p fhe-mir-71b-5p	Modulation of neuromuscular and metabolic transcripts Biosynthesis of exosomes and glycans	[72]
fhe-let-7a-5p fhe-mir-124-3p	Cell differentiation Stem cell regulation Growth induction Regulation of neuronal development	[77]
Bantam-3p	Downregulation of *F. hepatica* cathepsin L3 (FhCL3)	[18]
miR-1b-3p miR-71a-5p miR-71c-5p miR-125b-5p miR-190a-5p miR-277-3p	Nucleotide synthesis, transcription and translation Energy regulation Cell signaling Proteolysis	
miR-126a-3p miR-150-5p miR-155-5p miR-181a-5p miR-362-3p	Cell apoptosis Activity during parasite migration and proliferation	[79]

### 4.3. Role of microRNAs in the Immune Response During Host–Parasite Interactions

*F. hepatica* modulates the host immune response since NEJs enter the peritoneal cavity through the intestinal wall after the excystment of the metacercaria within the duodenum. These NEJs, together with their ESPs released, promote the development of M2-activated macrophages for 24–48 h post-infection [80,81], thus impairing the ability of innate immune cells to respond to stimulation and avoiding the development of antigen-specific Th1 and Th17 responses and enhancing the differentiation of Th2 and Treg cells. This scenario promotes infection and long-term parasitic survival within the host [82,83,84].

miRNAs identified within these ESPs play a role in predicted targeted sites related to the establishment of an immune response, thus suggesting its implication in the immunomodulation of the host’s responses, helping in the progression of parasitic infection [19,85,86]. Whether they are important for infection of the mammalian host has yet to be determined [17] and has been corroborated in response to stimuli across the microenvironment where it inhabits, suggesting immunoregulatory activity [34].

There are a number of miRNAs identified in research that stand out for altering the cellular and molecular mechanisms of the immune system in relation to host–parasite interaction. Concretely, fhe-miR-125b is internalized by host macrophages, mimicking host miR-125b and decreasing inflammatory cytokines by interrupting the Mitogen-Activated Protein Kinases (MAPK) signaling pathway through TNF-receptor associated factors 6 (TRAF6) targeting [35]. miR-125b in *Fasciola* spp. is closely related to its human homolog hsa-miR-125b, suggesting the implication in the control of host immune responses during early infection stages [35]. Previous studies have documented human hsa-miR-125b as a miRNA that regulates the inflammatory response of macrophages by controlling the activation of M1 macrophages [87,88], and its overexpression in host macrophages is related to an effective antigen-presenting cell stimulation of T cell responses, elevating the responsiveness to IFN-γ, thus suggesting a role for miR-125b in activating macrophages [89].

Moreover, the expression of miRNAs in macrophages in BALB/c mice infected with *F. hepatica* was also evaluated [90]. The expression of a total of 8, 22 and 3 miRNAs was registered at 6 h, 18 h and 5 days post-infection, respectively. These presented specific target genes related to the inflammatory response and induction of pro-inflammatory cytokines [91,92]. The expression of fhe-miR-125b was prominent within the parasitic miRNAs analyzed, corroborating previous research demonstrating its role within the molecular machinery of such protective cells [35,93]. Likewise, the total number of miRNAs present at 18 h post-infection was related to the migration of NEJs through the peritoneal cavity, a crucial time for their interaction with host-derived macrophages, suggesting the parasite’s need to evade a hostile environment until reaching the hepatic organ at approximately 5 days, where they crucially decrease [90] (Table 3).

It has been demonstrated in organisms such as *S. japonicum* that miR-125b and bantam interfere in host macrophage functions by increasing TNF-α, facilitating parasite development, metabolism and egg laying [94,95]. Likewise, the miRNA bantam has been implicated in the regulation of cell proliferation and apoptosis [96] and represents a direct marker for schistosomiasis in human serum [97]. Interestingly, Xue et al. 2008 [98] showed that three of the miRNAs that we find in serum (sja-bantam, sja-miR-71 and sja-let-7) are expressed during all the stages of parasite development but are enriched in the cercariae, suggesting that they may be important during the initial stages of schistosome infection.

In humans, there are 10 mature let-7 miRNAs, including let-7c [99]. It has been demonstrated that let-7c regulates bactericidal and phagocytic activities of macrophages, and the overexpression of let-7c in Granulocyte Macrophage Colony-Stimulating Factor (GM-CSF)-induced bone marrow-derived macrophages (GM-BMM) diminished M1 phenotype expression while promoting polarization to the M2 phenotype expression [100]. *F. hepatica* miRNAs may also have an interesting target: the *LIN28A* gene, which may minimize the host’s Let-7 miRNAs expression, thus helping in Treg differentiation [70,101] (Table 3).

EV-derived miRNAs perform a key role in the communication processes between parasite and parasite and host and parasite [102]. *Fasciola* spp. exosomes function as buffers at the interface of the cellular microenvironment for miRNA:mRNA matching, generating internalization of cells into their definitive hosts, disrupting the primary message transmitted to the cell, which provides a reduction in or distortion of their protective properties, leading to modulation of the immune system to ensure their migration through the intestine [35,72]. Specifically, miR-10 family members, bantam and let-7 family members have been postulated as important miRNAs implicated in the modulation of the host immune responses, being the most abundant miRNAs in EVs secreted by adult trematodes [38,103]. On the other hand, the miR-1 and let-7 family are part of the ESPs of EV in *Trichinella spiralis*, where they mediate the polarization of macrophages M2 and are related to the low regularity of the IL-10 and the activity of TGF-β in the host [104].

A significant part of the predicted targets for *S. mansoni* and *F. hepatica* EVs miRNAs are related to the glucose concentration in blood and surrounding tissues, T cell immunity and cytokine signaling. According to this, helminths may modulate the level of glucose concentration in host blood and tissue and reduce inflammatory and immune responses, and these modulations can contribute to the survival of the parasites inside their hosts [102].

Given the importance of macrophages during early and chronic immune responses in fasciolosis and the implication of miRNAs in its function, Bąska et al. 2024 [105] evaluated the expression of miRNAs in human macrophages THP-1 activated by lipopolysaccharide (LPS) derived from *F. hepatica* ESPs using microarray assays, but no evidence of internalized miRNAs in these cells was observed. miR-1537p was found to be the highly expressed miRNA but with low significance (Table 3). The authors attribute a low sensitivity to the detection of miRNAs by the microarray assay together with an insufficient concentration of *F. hepatica* ESPs. Previously, miRNA expression has been evaluated in *Fasciola* spp. infections involving the metabolism and response of mononuclear cells [35,106], where higher concentrations of ESPs were used. The analysis techniques involved next-generation sequencing (NGS), which is more commonly used and recommended for assertiveness than microarray [107].

The capacity of *F. gigantica* ESPs to dysregulate hepatic miRNAs in mice and to change the expression of miRNAs in goat PBMCs has been previously reported [79,106]. In the 2021 study of Wang et al. [106], a reduction in the expression of miR-211 and miR-204-5p was reported, which may imply a possible involvement of *F. gigantica* ESPs in promoting the proliferation of PBMCs. The expression of miR-148a-5p was upregulated, and this has been described to inhibit pro-inflammatory cytokines and may help in shifting Th1/Th2 towards the Th2 immune response [106].

In ruminants infected with *F. gigantica*, a dysregulation of circulating miRNAs was observed, and four parasite-derived miRNAs (fgi-miR-87, fgi-miR-71, fgi-miR-124 and fig-miR-novel-1) were detected in serum, indicating that the host’s circulating miRNA profile is altered during *Fasciola* infection [108]. Concretely, miR-71 was evidenced in the exosome content in *Brugia malayi* and *F. gigantica* [108,109], which mediate the nitric oxide levels on host macrophages [110] and modulates the host immune response together with miR-87 [103]. In addition, miR-71 and miR-87 have been suggested as biomarkers in fasciolosis [108]. Robb et al. 2022 [77] described a strong downregulation of fhe-mir-124-3p in in vivo *F. hepatica* juveniles, suggesting its connection with neuronal cell differentiation and contributing to the control of numerous biological processes.

Apart from those, miR-126a-3p, miR-150-5p, miR-155-5p, miR-181a-5p and miR-362-3p were found to be dysregulated in the liver of mice treated with ESPs from *F. gigantica* at 1, 4 and 12 weeks post-exposure, revealing their important roles in the interplay between the liver and *F. gigantica* ESPs. Interestingly, all these dysregulate miRNA participate in cellular apoptosis, migration and proliferation, suggesting that they may play roles in liver pathological changes induced by the liver flukes [79]. In addition, the downregulated expression of miR-466i-5p and miR-423-5p may be implicated in biliary secretion and liver functions in *F. gigantica* infection, and high expression of miR-466-5p has been found in hepatocytes in response to inflammatory stimuli [79,111]. The expression of miR-155 has been related to chronic liver inflammation and fibrosis, and miR-126 plays a role in increasing the inflammatory activity in the liver while inducing the expression of IFN-γ, MAP kinase pathways and differentiation of FoxP3 regulatory cells [112,113].

**Table 3:** Activity of microRNAs (miRNAs) in the immune response during host–parasite interaction in fasciolosis.

miRNAs	Pathway	Reference
fhe-miR-125b	Internalization of macrophages by mimicking host miR-125b	[35]
Bantam	Regulation of cell proliferation and apoptosis Modulation of host immune responses	[38]
Let-7	Treg cell differentiation	[70]
miR-211 miR-204-5p	Peripheral blood mononuclear cell reduction	[106]
miR-148a-5p	Inhibition of pro-inflammatory cytokines Dysregulation of Th1/Th2 to Th2 immune responses	
miR-71	Regulation of nitric oxide levels in macrophages	[108]
miR-466-5p	Response to inflammatory stimuli	[79]

### 4.4. microRNAs as Biomarkers and Potential Therapeutic Targets

Another point of interest in *Fasciola* spp. miRNAs is their importance as promising biomarkers for disease diagnosis as well as novel targets for therapeutic intervention [19,114]. Concretely, miR-124, miR-87 and miR-71 have been previously described in fasciolosis and postulated as promising biomarkers for this disease [71,108], as well as miR-277 has been recognized in host serum [97,115], where it is involved in the regulation of enzymes that play a role in the survival of the parasite under stress or starvation conditions [116] (Table 4).

The sheep miRNAs postulated as potential biomarkers in pre-hepatic infection were oar-miR-133-5p, oar-miR-232a-3p and oar-miR-1197-3p, being oar-miR-3956-5p consistently elevated both in pre-hepatic and hepatic infections [20]. These miRNAs have been evidenced in other types of diseases where they have also been postulated to act as possible biomarkers [117,118]. Chowdhury et al. 2024 [20] evaluated the potential of miRNAs present in sera as potential biomarkers for the early diagnosis of fasciolosis in sheep. Two parasite miRNAs (miR-124-3p and miR-Novel-11-5p) were postulated as potential biomarkers as they were detected at all time points during *F. hepatica* infection.

In addition, two mature miR-1992 and miR-novel-3 sequences could be of interest to differentiate the two fluke species [71] (Table 4). This could be of interest for their use as taxonomic markers as *F. gigantica* and *F. hepatica* can co-infect the same host, increasing the virulence of the infection mainly due to the development of anthelmintic resistance scenarios [71], where it has been reported that altered expression of miRNAs may be associated with such resistance to the antiparasitic drugs used to control them [119].

This is why, just as the miRNAs, long non-coding RNAs (lncRNAs) have been mainly associated with transcriptional regulation. The first description of the lncRNA complement of *F. hepatica* was made by McVeigh et al. 2023 [120]. lncRNAs contain miRNA binding sites as demonstrated by the in silico binding prediction between the 150 miRNAs previously reported in *F. hepatica* [18,72] and the lncRNAs described in *F. hepatica* [120], resulting in a consensus set of 4104 lncRNA:miRNA pairs. The large amount of miRNA binding sites on lncRNAs may indicate a miRNA-driven post-transcriptional regulation of lncRNA expression. The hypothesis that lncRNAs contain miRNA binding sites and thus act as “sponges” for miRNAs has been previously exposed [121]. Alternative therapies for the treatment of fasciolosis could be derived from the implementation of silencing these lncRNAs or interrupting the regulatory interaction with its target similar to miRNAs, which are currently under study in many clinical trials to become an alternative means of therapy for many conditions [120,122].

## 5. Unraveling the miRNome in Fasciolosis

Since the first evidence of miRNAs for *Fasciola* spp., characterization of candidate families has been achieved through various methodologies with a greater focus on *F. hepatica* than *F. gigantica*. The first five investigations [34,67,68,69,70] gave a total of 85 miRNAs for *F. hepatica*, but 8 of these [34] were claimed to be genomic repeats [67,68], leaving a total of 77 conserved, where 36 are expressed in the juvenile and adult stages, 15 appear to be exclusive in NEJs and 26 in the adult, considering 17 as novel as they do not match counterparts in other organisms [37].

The number of miRNAs for *F. gigantica* is less clear. To date, ~92 miRNAs have been published throughout its life cycle, where ~47 appear conserved with *F. hepatica* [34,71,106,108], postulating miR-1992 and miR-Novel-3 which could differentiate the two strains of Fasciolidae, ~12 are homologous with other species [71], and ~33 are suggested to belong to conserved families. However, since their sequences are not presented in the publications or in the supplementary material, it is difficult to confirm this hypothesis.

Ricafrente et al. 2022 [18] expanded the *F. hepatica* miRNome to 124 miRNA among NEJs, immature (12 dpi) and adult stages, of which 72 were previously reported and 52 were discovered with 3 of these as conserved sequences (fhe-miR-493-5p, fhe-miR-2335-5p and fhe-miR-6613-3p), and 49 were assigned as novel, with 31 miRNAs homologous to *F. gigantica* and 18 specific to *F. hepatica*. At the same time, Fontenla et al. 2022 [38] confirmed the miRNAs previously identified in *F. hepatica* by Ricafrente et al. 2021 [37], validating four of them as homologs of the miRNAs described for *F. gigantica* [71] and adding nine miRNAs classified as novel (fhe-miR-NEW-1 to 9) present in all stages of the parasite and its EVs, suggesting that seven sequences are conserved within *F. gigantica*.

A manual reset of the miRNAs published for *F. hepatica* previously [34,67,68,69,70] obtained a set of 89 non-redundant miRNAs conserved for the worm, including 3′ and 5′ and precursor hairpin regions [72]. The sequence alignment performed expands the number of specific miRNAs to 52 (fhe-novelmir-1 to 45), maintaining the expression of 3′ and 5′ regions of mature miRNA sequences that manifest it, leaving a total of 150 miRNAs present at different stages of their life cycle. The presence of a large number of novel miRNAs suggests the evolution of these ncRNA, inviting the discovery of homologs in *F. gigantica* that support this hypothesis [72].

Thus, in 2022, three studies were published adding 70 new sequences for *F. hepatica* [18,38,72], but two of these, named fhe-Novel-3 and fhe-Novel-39, were reported by Ricafrente et al. 2022 [18] as identical to two miRNAs (fhe-novelmir-44-5p and fhe-novelmir-18-5p) described by Herron et al. 2022 [72], suggesting that the current number of novel miRNAs is 68. However, Herron et al. 2022 [72] performed sequence alignment taking into account the 3′ and 5′ regions that a single miRNA might have, which may suggest divergence between mature sequences of the same miRNA [52]. In addition, Herron et al. 2022 [72] gave a consensus name to the 150 miRNAs using the nomenclature for the annotation of miRNAs in databases [73]. This would avoid the redundancy generated by different authors, thus allowing miRNAs to be precisely identified. The last two publications [77,120] that integrate miRNome into their study refer to the report by Herron et al. 2022 [72], making it possible for the miRNome to support 150 miRNAs in total.

**Figure 3:**
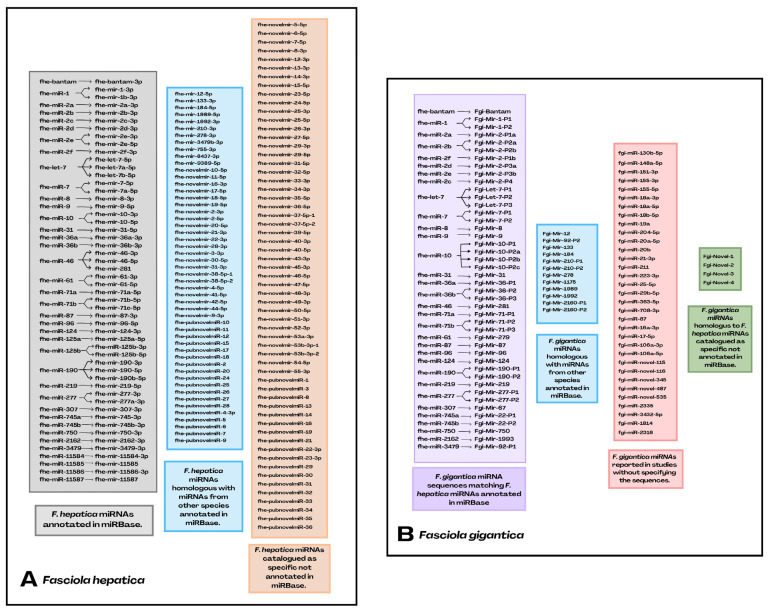
Overview of the miRNome in *Fasciola* spp. based on the latest miRNome reports for *Fasciola hepatica* [72,77] and *Fasciola gigantica* [71] displaying the nomenclature based on the annotation of microRNAs (miRNAs) in databases [73]. (**A**) represents the 38 miRNAs annotated in miRBase for *F. hepatica* with the 3p/5p sequences reported to date (gray), the homologous sequences with other species/organisms annotated in miRBase that are not annotated for *F. hepatica* (blue), and miRNAs without homologous sequences to any of the miRNAs from other species/organisms annotated in miRBase, considered as specific for *F. hepatica* without annotation in miRBase (orange). (**B**) represents *F. gigantica* miRNAs homologous with miRNAs annotated for *F. hepatica* in miRBase (purple), miRNAs from *F. gigantica* that have homology with miRNAs from other species/organisms annotated in miRBase (blue), miRNAs reported within the *F. gigantica* studies without sequences to help identify them (red), and *F. gigantica* miRNAs with homologous sequences in miRNAs specific for *F. hepatica* without being annotated in miRBase (green).

Finally, Robb et al. 2022 [77] identify 14 new miRNAs for *F. hepatica* with supported mature sequences, classifying them as novel and listing them under the previously stipulated consensus name (fhe-novelmir-45 to 53) and retaining the 3′ and 5′ regions of the miRNAs that presented it, leaving a total of 164 miRNAs for the worm at present. However, the miRNA sequences accepted to date in miRBase are 38 for *F. hepatica* and considered to be genuine to one sequence, but others have not been added because they do not meet the classification criteria for miRNA formation and maturation, which possibly refers to the type of precursor hairpin assembly and the assertiveness of the analytical tools used [73]. Nevertheless, the criteria in other databases, such as mirGeneDB, consider a wider range for hairpin type and expression of miRNAs according to the variety in their canonical/non-canonical biogenesis, DROSHA- or Dicer-dependent, change at the 3′ and/or 5′ ends postulating further criteria for the annotation of miRNAs in metazoans [37]. For this reason, it is suggested that the number of miRNAs that researchers describe in their studies within the miRNome be taken into account, even if they are not yet included in the miRBase (Figure 3).

## 6. Conclusions

To date, the discovery of miRNAs has significantly enhanced our understanding of fasciolosis. The prominent focus in most published research highlights miRNAs such as miR125b, let-7, bantam, miR-10, miR-124, miR-87, miR-71 and miR-277 in key aspects of the disease, including the growth and development of its strains throughout their life cycle, as well as pathogenesis and their interaction with the host for parasite survival. In addition, the innovation of miR-1992 and miR-novel-3 may help differentiate between the two strains of fasciolosis, leading to more effective methodologies for studying each worm. This could yield better insights into the molecular networks involved, ultimately identifying critical points for controlling the disease to benefit both human and animal health. Furthermore, generating an updated and standardized miRNome is essential for researchers, providing easier, more organized and comprehensive access to all functional miRNA files related to *Fasciola* spp. infections. This will pave the way for new studies that integrate multi-omic data, allowing for a holistic view of biological systems and leading to the identification of new biomarkers and therapeutic targets.

## Figures and Tables

**Table 4:** MicroRNAs (miRNAs) considered as biomarkers in the diagnosis of fasciolosis.

miRNAs	Pathway	Reference
miR-124 miR-87 miR-71 miR-277	Overexpression in *Fasciola gigantica* infections	[71,108]
oar-miR-133-5p oar-miR-232a-3p oar-miR-1197-3p	Potential for circulating activity during the diagnosis of pre-hepatic and hepatic fasciolosis in sheep	[20]
miR-124-3p miR-Novel-11-5p	Overexpression sustained during infection with *Fasciola hepatica*	
miR-novel-3	Mostly related to *F. hepatica*	[71]
miR-1992	Mostly related to *F. gigantica*	

## Data Availability

No new data were created or analyzed in this study.
